# Our Right to the Pleasure of Falling in Love

**DOI:** 10.3389/fpsyg.2019.03068

**Published:** 2020-01-22

**Authors:** Elisabeth Torras-Gómez, Lídia Puigvert, Emilia Aiello, Andrea Khalfaoui

**Affiliations:** ^1^Department of Sociology, University of Barcelona, Barcelona, Spain; ^2^Kennedy School of Government, Harvard University, Cambridge, MA, United States; ^3^Faculty of Psychology and Education, University of Deusto, Bilbao, Spain

**Keywords:** coercive dominant discourse, attraction to violence, hooking up, romantic relationships, social impact

## Abstract

The social impact of psychology on the field of human sexuality is extensively wide. From Freud to Masters and Johnson, many are the research which have broken barriers and provided citizens with new knowledge to improve their lives. One of the lines of research which are now contributing to this social impact from psychology is that of the dominant coercive discourse (Gómez, 2015), which portrays power relationships as exciting and egalitarian relationships as convenient. Drawing from this theory, the aim of this research is to shed light on the influence of the coercive discourse on women’s pleasure in their intimate relationships. In an exploratory study, women between 20 and 29 years old were interviewed under the communicative methodology. Results show three main findings. First, participants who reject the coercive discourse find pleasure in egalitarian relationships. On the contrary, participants who had coerced relationships acknowledge a lack of excitement in egalitarian relationships, while associating pleasure to the power nature of the former. Finally, some participants who initially had coerced sexual–affective relationships were able to disassociate pleasure from coerced relationships and break with them. Moreover, these women claim to feel more pleasure in their new egalitarian relationships. These findings open a new path of research that unveils the lack of pleasure in coerced relationships and vindicates our right to the pleasure of falling in love.

## Introduction

Psychology has had a wide social impact on the fields of human sexual behavior and sexual desire. Advances in the field of psychology have demonstrated that, besides biological or even sociological factors, sexual desire depends as well on psychological ones.

One of the first authors to explore the topic was the psychoanalyst Freud. Freud (2016) focused on sex as the main element in human development, since he described libido as the force driving human behavior. Through his psychosexual development theory, he described five stages which humans follow in their lifespan: the oral stage, the anal stage, the phallic stage, the latent stage, and the genital stage. According to him, these were determinant to human development: failing to successfully pass them could lead to psychological problems and mental disorders. Even if psychoanalysis is now being questioned because of the difficulties to evaluate this theory following a scientific methodology (Kandel, 2018), its contributions to the exploration of human sexuality and human behavior regarding sex cannot be denied.

In the late 1940s, Kinsey (Kinsey, 1998; Kinsey et al., 1998) started conducting large-scale surveys of the American population’s sexual activities published. For the first time, his reports provided evidence of sexual behavior of humans, including frequency, practices, and lifestyle, among others. However, the information gathered in these reports about sexual behaviors remained statistical.

Indeed, it was not until the research of Masters and Johnson that the first evidence of how humans experience sexual arousal and sexual activity arrived. Even if now ethically controversial, the research conducted by Masters and Johnson regarding human sexual response (Masters and Johnson, 1966) and human sexual inadequacy (Masters and Masters, 1980) are considered among the 40 studies that changed psychology (Hock, 2001). These researchers explored the physiological responses in human sexuality, which they saw as fundamental for a satisfying sex life. Masters and Johnson complemented these works with a series of books in which they explored the psychological aspects of sexuality. Their work continues to influence scientific research on human sexuality in several fields, including psychology.

More recent research keeps challenging the reproduction theories by bringing forward evidence of pleasure being one of the main variables that explain sexual motivation (Meston and Buss, 2007; Barnett and Melugin, 2016). Indeed, hooking-up has been associated with physical pleasures (Farvid, 2014), such as stress and tension relief or fun, as well as with psychological and affective pleasures, including ego boosting and thrills linked to mischieving, transgression, and novelty (Farvid and Braun, 2017). Often these casual sex experiences involve alcohol intake (Claxton et al., 2015), which Pedersen et al. (2017) link to pleasure derived from control loss, time-out from normative life and recounting it to friends as “a crazy and wild experience.”

However, research also shows that women associate hooking up with high regret (Campbell, 2008) and disgust (Al-Shawaf et al., 2018; Kennair et al., 2018) after engaging in casual sex, while those in a relationship report higher levels of pleasure than those who engage in casual sex and highlight the importance of care and love for “good sex” (Paik, 2010; Carlson and Soller, 2019). According to different papers, the negative feelings could be due to multiple and inconsistent reasons such as impelling sexual motivation (Campbell, 2008) or sexual double standards (Armstrong et al., 2010; Snapp et al., 2015; Rodrigue and Fernet, 2016; Farvid and Braun, 2017; Uecker and Martinez, 2017), and some point out to the negative outcomes of long-term relationships, such as controlling and violent partners (Armstrong et al., 2010). Nevertheless, scientific research has already provided evidence which shows that positive or negative outcome in a relationship do not depend on its length, but on the partner of choice (Puigvert et al., 2019).

In this vein, another theory which has contributed to the social impact of psychology is that of the coercive dominant discourse (hereinafter, CDD). Gómez (2015) argued that the traditional model of partner election which links sexual attraction to domination, imposition, and contempt is one of the socializing elements that influence the association of passion with suffering, while more egalitarian values are seen as convenient but lay far from desire. This traditional model is conveyed through the CDD in numerous daily interactions with peers, TV shows, popular songs, and social media, among others.

This continuous presentation of men with violent attitudes and behaviors as attractive progressively socializes some women from a young age into attraction toward violent attitudes and behaviors. Indeed, novel research on socioneuroscience has pointed out that the frequent association of violence to attractiveness is internalized by some women, leading them to feel aroused before men that present violent characteristics (Puigvert Mallart et al., 2019). As the authors explain, this emotional reactions are not in fact their own, but the consequence of the socialization in the pressures of the CDD that emerges from the power imbalance within relationships fostered by our patriarchal society. In line with these findings, many studies report girls to prefer partners with aggressive features for hooking up (Valls et al., 2008; Puigvert et al., 2019). These girls say to prefer “good guys” for when they get established in relationships, while they rather choose the bad ones, the fun ones, for short stands (Gómez, 2015). Nevertheless, preferring this type of guys puts them at a greater risk to suffer intimate partner violence.

Alongside, as part of this discourse, girls and women are told to break with alleged “pressures” which force them to save their virginity. However, the peer pressure conveyed through the CDD is also related to what has been defined by existing literature on gender violence prevention as the “upward mobility mirage” (Oliver, 2010–2012). When girls and women fall victims of this process of upward mobility mirage, they think that having intimate relationships with boys and men with violent attitudes and behaviors, they will move up in the social chain. However, what actually happens is the opposite: girls who hook up with many “bad boys” are less socially valued by their male and female peers. Therefore, gratification and pleasure in these cases seems to be related to the perceived social status. In addition, several studies show that students who felt pressured by their peers to engage in casual relationships seemed more susceptible to adverse outcomes related to hooking up, as well as were those hooking up with multiple partners (Montes et al., 2016, 2017).

The notion of romantic love has traditionally included inequality between men and women but at the same time love and respect and not violence at all. Currently there is a feminist transformation of this concept which maintains the non-violence but overcoming its inequalities. However, at the same time, there are other transformations of the concept which maintain inequalities and include violence in romantic love (Lelaurain et al., 2018). The feminist transformation of the concept allows girls and women to choose if they want to look for romantic love free of violence with other girls or boys. The other transformation of the concept prevents women to have that kind of love while it pushes them to sporadic relationships which often include more violence than stable ones. Indeed, there is no evidence of romantic relationships under these terms leading to gender-based violence (Yuste et al., 2014).

Drawing from the idea that pleasure is one of the main reasons to engage in sexual relationships, in the present study we explore the effects of the CDD on the pleasure of young women. The aim is to provide evidence on how CDD influences the partners they choose, the type of relationship they have, the sexual pleasure they associate to them and, on the long-term, what they expect from a relationship and partners (sporadic or stable). On the other hand, we also want to explore if these preferences are different in young women who have not been victims of these pressures. We hypothesize that pleasure in relationships is not related to the duration of those (long or short-term, sporadic, and stable) or to sex, but to dominant and coercive social preferences regarding the type of partner and relationship (coercive vs. egalitarian) which have been internalized through socialization. Conversely, we expect love to be a protective factor against the CDD. With our results we expect to extend the social impact of psychology by providing some answers that allow to move forward toward violence-free and consented sexual–affective relationships (Vidu Afloarei and Tomás Martínez, 2019).

## Materials and Methods

### Design

The study follows the communicative methodology of research (CMR). This approach pursues the transformation of social realities through the egalitarian dialog and the inclusion of all voices (Gomez et al., 2019). In the CMR, researchers share their scientific knowledge with the researched subjects, who in turn contribute with their own knowledge and experiences of the social reality which is being explored. This is possible thanks to the establishment of an egalitarian dialog that allows to overcome the relevant gap between scientists and researched subjects (Habermas, 1987) and to provide new solutions to the problematics being discussed. The implementation of CMR has contributed to the social impact of psychology regarding violence prevention (Oliver, 2014).

### Participants

Thirteen young women (P1–P13) from different socioeconomic backgrounds and geographical regions within the Spanish context were recruited for the study, using purposive and snowball sampling. No gatekeepers were used. All of them were between 20 and 29 years old and all of them reported having had heterosexual relationships. Twelve of them had completed a bachelor’s degree and one of them was in the process of doing so. Eight of them had also completed a master’s degree and two of them were currently in the process of finishing it. No participants withdrew from the research after signing the consent form.

### Materials and Procedure

The current research was fully approved by Community of Researchers on Excellence for All’s (CREA) Ethics Committee. It complies with the European Commission’s Ethics Review Procedure (2013), the Data Protection Directive 95/46/EC, and EU’s Charter of Fundamental Rights (2000/C 364/01). Before being involved in the research, participants were contacted individually by the researchers, who fully informed them about the study. They were given a written “informed consent” in which the specifications of the study were detailed: scientific background of the proposal, aim, methods, and procedure. Participants were given time to read it and were told that they could ask any questions at all times. The researchers gave clarifications when necessary. Participants were also informed about their possibility to withdraw at any time from the study. Taking the intimate content of their testimonies, they were granted full anonymity and their identities were concealed from the beginning of the research. Due to the nature of their responses, they could decide if they wanted to be audio-recorded or if they preferred the researcher to take notes on their statements. Once the results of the study were ready, they were sent to the participants in order to ensure their conformity with publication.

A semi-structured interview was conducted with each participant individually. The questions for the interview were designed by consensus between all researchers. Knowledge from previous studies regarding the CDD and its influences on relationships and partners was considered when creating the interview. Questions were arranged temporally, from childhood to present. Finally, the script was composed by 33 open-ended questions and subquestions about their sexual–affective relationships, their own feelings, and behavior about them, as well as that of their peers. Interviews lasted from 50′ to 1h15′. Each testimony was either audio recorded or gathered through the researcher’s notes, according to the participant’s will.

### Data Analysis

The current study follows the saturation criteria of Guest et al. (2006), according to which these 13 interviews allow for reaching theoretical saturation since participants were purposively selected; besides location and SES, the group was relatively homogeneous and the domain of inquiry has been delimited. The participants’ narratives were analyzed as communicative acts (Searle and Soler, 2004). This approach considers the role of both verbal and non-verbal communication, it separates the intentions behind the acts from their consequences, and accounts for existing power relations in the social context of the speakers. The approach has already proven successful at better identifying situations of coercion, while providing elements of analysis for overcoming difficulties and transforming realities (Rios and Christou, 2010). Under this approach, the gathered testimonies were analyzed following a line-by-line technique. For the early coding, the content of their testimonies was classified into three temporal categories: childhood, adolescence, and early adulthood. Within each category, testimonies were scrutinized for evidence regarding the type of relationship and partner preferred, their own behaviors, feelings, and attitudes toward, as well as that of their peers. Discrepancies between the researchers concerning the data coding were sorted out by consensus. For these cases, researchers discussed the interpretation of each fragment and collectively decided its categorization.

On a second review of the categorization, the following elements of the CDD were identified (Table 1).

**TABLE 1:** Elements of the CDD.

Elements	Description
e1. Peer pressure	How have the ideas and preferences in their context influenced them?
e2. Partner and relationship choice	How and why did they choose their partners and relationships?
e3. Coerced relationships and partners	How are coerced relationships and partners described and remembered?
e4. Fake narratives	Have they justified bad experiences or share them as exciting to meet social expectations?
e5. Transformation	Is there a change of view regarding intimate relationships preferences and the feeling of pleasure over time and experiences?
e6. Egalitarian relationships and partners	How are they described and remembered?
e7. Right to pleasure	What do they find exciting and/or remember as exciting?

The analysis of the participants showed certain patterns in the appearance of the aforementioned elements, which had an impact on the way participants understood and perceived pleasure. The three models and their characteristics and differences will be presented in the following section.

## Results

As a result of the analysis, a series of elements were identified in the speech of participants. The way in which these elements appeared and converged allowed the identification of three different models, regarding how participants have chosen and choose their partners and relationships, as well as the impact this has on the way they understand and experience pleasure. These elements result from the CDD conveyed through multiple interactions in the context of a patriarchal society. They reflect the effects of such discourse on young women’s partners of choice and relationships and the pleasure they associated to these. Table 2 presents the distribution of the participants in each model.

**TABLE 2:** Distribution of participants in each model.

	P1	P2	P3	P4	P5	P6	P7	P8	P9	P10	P11	P12	P13
Model 1	X	X											
Model 2			X	X	X	X	X	X	X	X			X
Model 3											X	X	

### Model 1 – Not Giving Up the Right to the Pleasure of Falling in Love

When participants were first asked about their childhood, they were suggested to describe their ideal partner and their ideal relationship; to share what these would entitle. Participants classified into Model 1 (M1) described positive and egalitarian relationships as it can be seen below:

“*Very romantic, like in Disney movies. a person who is always, always there, someone emotional, romantic* … . *someone who fits in my family, funny, loving, who takes care of me. Someone with whom I have a good time, outgoing*.” (P1)

In this extract we can see that M1 participants think that an egalitarian relationship can have both love and passion. They also describe their partners through the language of desire, that is the type of language to express admiration, attraction, and desire (Rios-González et al., 2018), with words such as “loving” and “romantic.”

However, participants in M1 explain that during adolescence the view of relationships which was socially shared among their peers had nothing to do with their own ideal. In fact, they acknowledge having experienced *peer pressure* (e1) to engage in other type of relationships and partners. However, they also state that even if what their friends did was important to them and that they felt curious about it, they did reject these pressures. An example of this can be read below:

“*I didn’t experience direct pressure, but indirect yes. Since others were doing it you, you felt the desire to do it as well* … *They used to tell me ‘but don’t you get bored? You’re very young, you have to try more* …*’ And if I don’t want to, what? It has been very clear to me from the beginning*.” (P2)

In this extract P2 describes how those in her group of friends kept questioning her choices and pushing her to try something different. However, her ideal of a relationship and partner was so clear to her that she did not want to give it up and remained strong before these pressures. Accordingly, regarding *partner and relationship choice* (e2), participants in M1 describe to have chosen and still choose their partners and relationships based on their own convictions. In their testimonies, there is a clear rejection to being with someone for any other reason than love and attraction. As P2 explains:

“*Sporadic relationships have never attracted me, going to a house to have [sexual] relations with a stranger, with a stranger? That has never excited me. I do not get excited with a stranger. I have to like previous things*.” (P2)

In the same vein, when talking about *coerced relationships* (e3), M1 participants share strong negative feelings about them. They explain how some of their friends ended up with boys without liking them or with boys who did not treat them well, just because they were socially valued. However, even if they understand the reasons behind their friends’ actions, they recognize a lack of pleasure in such practices and express it this way. As P1 explains:

“*There were people around me who did things because they felt they had to, especially in sex. Maybe at that time they didn’t give the same value to what they did and what they felt. To me, both have always been equally important* … *I shared what I felt and what happened to me. With some friends I have doubted that they were telling the truth. for whatever reason, to please more, because of what others may think*.” (P1)

In addition, as it can be seen in the extract, M1 participants acknowledge to be aware of the fact that some of the things their friends were sharing regarding these relationships were not true; they were telling them in order to meet social expectations. Moreover, they also recognize that sharing these *fake narratives* (e4), in which they did not feel the need to engage, has led their friends to end up liking such coerced relationships and partners who mistreated them. This is the situation described by P2:

“*In this girl’s case* [a friend]*, she gave in to the pressure and it was not a good experience for her. She wanted to like the boy, even if she was disgusted by him, and then she ended up liking him*.” (P2)

Therefore, taking into account both their ideal of a relationship as children and this same ideal now, no *coercive transformation* (e5) is perceived in M1 participants, rather a continuous preference toward egalitarian relationships and partners. As seen in this subsection, M1 participants have preferred this latter type of relationship throughout their life, even when other type of relationships was socially valued by their peers. In addition, they explain to have chosen egalitarian relationships over coerced ones, because they understand that pleasure is a core component of the former, while the latter completely lack it. In this vein, M1 participants share that they choose *egalitarian relationships and partners* (e6) because according to them they unite both, ethics and desire. As P1 shares:

“*In my case, as I have been with my partner for so many years, I can say it’s the best memory I have, both emotionally and sexually* … *My relationship is passionate because of what it makes me feel. It makes me feel great in all physical and emotional aspects. it moves me. To me that is passionate*.” (P1)

Through this example we see how M1 participants have not given up their *right to pleasure* (e7). In fact, they believe pleasure is linked to falling in love and freedom. They feel they have passionate relationships in which they share tenderness and excitement and that these are feelings they have built their relationship upon, making it ideal to them.

### Model 2 – When the Coercive Dominant Discourse Steals the Right to the Pleasure of Falling in Love

When asked about their ideal partner and their ideal relationship, participants classified into Model 2 (M2) also described egalitarian relationships with handsome and brave partners:

“*I imagined it as what it is seen on TV, in movies, that everything is ideal, everything is going great, boy and girl in love, all beautiful, they have no problems, and then you see that it’s a lie, obviously*.” (P8)

As seen in this example, participants in M2 mainly dreamt too with idyllic relationships in which there is love and everything works out. However, a feeling of deceit can be perceived in the words of some of the participants in M2 regarding this ideal of relationship they had as children. In this vein, one can see how P8 points out that her former ideals were naive and unrealistic. She feels everything is “a lie”; these ideals are something unachievable to her.

When looking at what happened during adolescence, most M2 participants describe strong *peer pressure* (e1) in their context, even if they are not directly aware of it:

“*There was a time in which if you didn’t have a boyfriend or weren’t hooking up with someone, you were considered a loser. I started late* … *The one who was with the most handsome guy, with the hottest, was the most socially valued* … *At that time, I don’t believe that having a stable relationship was more valued* … *the one hooking up with more guys was more socially valued.*” (P3)

In this extract P3 recognizes that not falling into the pressures of the CDD had negative social consequences, which is the reason young women in M2 fall into them, as we will see in this section. Along the same line, most young women in M2, like P3, mentioned that they believed that the more you hooked up with the cool guys, the more your social status increased. This fact has highly affected M2 participants’ partner and relationship choices (e2). In their narratives, these young women share that they have primarily chosen those partners and relationships which were valued in their social groups, sometimes even when they knew it was not something they wanted:

“[The guys with whom to hook up were] *the indifferent, the bad boys. They had to be handsome in the eye of others, yes* … [about hooking up with them] *Everyone was doing it, otherwise I wouldn’t have done it. It was what had to be done and almost everyone in my group did it. It was important to do it, not what you felt, because if you were going out and didn’t do it on the next day you were devastated.*” (P6)

In this extract P6 clearly acknowledges that the partners they chose had to be accepted by their social group. This is a common characteristic for young women in M2, who fell into peer pressure in order to fit in and keep a status within their group, as commented above. Another fact that can be observed in this extract is related to the type of relationships they engage in. In this case, we see how P6 recognizes that what was important was doing what was expected from you, beyond your liking or your feelings. Indeed, she acknowledges she would not have done it if everyone else was not doing the same and, at the same time, she shares that not engaging in such practices had negative social consequences. In the same way, this evidence shows how M2 participants, because of the CDD, end up in *coerced relationships* (e4). Regarding these, another common characteristic of M2 participants is that they share ambivalent feelings related to the coerced relationships in which they engaged and the coercive partners they choose:

“*I told it as if it had been very cool, but in reality, I had experienced anxiety in a boy’s house, but then I came back. why did I repeat [with him] if I felt anxious, why did I go back to his house? With my friends it was more like ‘wow, I’ve done this, I’ve done a bad thing’ It was about doing something malicious, a mischief.*” (P10)

The ambivalence is clearly present in P10’s testimony. She acknowledges having a bad time, feeling anxiety, but repeating this behavior she considered “bad” because she then felt excited about telling her friends, even though she knew it was not true: “*the next day you were devastated*”. In fact, fake narratives are another common element to most young women in M2. In their testimonies, these young women often acknowledge a lack of pleasure in the coerced relationship in which they engage, but end up justifying their actions or sharing them as exciting, in order to meet social expectations:

“*I was doing it wherever and before doing it I wanted to, but later I regretted it because I thought ‘what a drag!’. Doing it like that, one night. I did not enjoy it. To me, doing it like that was not like ‘wow, how cool’; rather, it was like eating an expired yogurt. People sell it like it’s amazing, but they do not feel it that way. I think it’s a lie, it’s to fit in. It’s like when you go on a trip and you say it’s been amazing, but it’s really been a fucking shit*.” (P6)

Both the lack of pleasure and the fake narratives are mentioned in P6 testimony. She acknowledges that she was not forced to do anything she was not choosing, but she did not feel like doing it either. In fact, she describes those relationships as “being a drag” and compares the encounter with “eating an expired yogurt,” which is something clearly unpleasant. In addition, she acknowledges to lie about it in order to fit in and she believes everyone around her participated in the elaboration of these lies.

Therefore, considering their childhood ideal of a relationship, together with their life experiences and how they share them, a *coercive transformation* (e5) is identified in participants in M2. Looking at the evidence presented this far, a negative shift is perceived: they have given up their ideal of a relationship and fallen into coerced relationships and partners. In addition, when looking at how they talk about their current relationships, M2 participants fall into double standards. In the same spirit, *egalitarian relationships and partners* (e6) are described through the language of ethics in their testimonies:

[talking about the best and the most exciting] “*they are completely opposite.* [The best one, the current one, is] *healthy, public, close, routine, status, couple, economy between two people, fixed, comfortable. The other one* [the most exciting] *is natural, uncontrollable emotions, was not forbidden but out of context, having a partner, was a teacher, we did not fit. there was the tension factor, tension to think that we had such a strong connection, ignorance, lack of control, disinformation, desire, sexual tension when it is not easy. They break what you’ve learned, surprises come, taking off, more instinct.*” (P7)

In this extract we see how even if saying that the best relationship is the current one, her description lacks excitement, as she continuously describes it in terms of “convenience”: status, economy, comfortable. This characteristic is common between participants in M2, who consciously or unconsciously end up choosing nice partners who treat them well. However, as seen before, there is a lack of transformation because they still identify those relationships in which they recognized coercion and a lack of pleasure as even more exciting than their best relationship. This leads to the last identified element shared by young women in M2. Regarding pleasure, this element is not a key component of the egalitarian relationships they now say to prefer. Similarly, some participants, like P7, pretend to fill this void by cheating on their partners, while others, like P6, consciously or unconsciously keep separating ethics and desire:

[talking about their most exciting relationship] “*just before being with him* [current boyfriend]*, I had a boyfriend. He was older. I met him on twitter. It was very cool on twitter; typical platonic love. I could not imagine getting to be with him, but then it was a fucking shit. At first it was exciting, then it was shit. At first, I did not know him very well. At the beginning, with the other I had to fight for him. I wondered about things, but with [my current boyfriend] it was more tender. He wanted to get to know me, he was very good, and he wanted to protect me. If I had to choose, I would choose this as the other was a fantasy.*” (P6)

P6 was the girl who described coerced relationship as “eating an expired yogurt” and she now acknowledges she now has a good boyfriend by her side. However, when asked to talk about her most exciting relationship, she decides to share one that was very tempestuous and, even if in the end she says that she prefers her current one, she finishes by saying that the former one was a “fantasy.” This shows that pleasure is still subject to the coercive discourse to her, since a single partner cannot unite both love and passion. Thus, the evidence presented this far shows that young women in M2 may have given up their right to the pleasure of falling in love.

### Model 3 – Taking Back the Right to the Pleasure of Falling in Love

When asked about their childhood ideal of a partner and relationship, participants classified into Model 3 (M3) also mentioned egalitarian relationships and partners, which they describe through the language of desire:

“*A prince who treats you well, helps you when you’re in trouble, based on love. The brave boy, attractive, nice, who doesn’t speak badly or treat you badly*.” (P11)

As this extract shows, young women in M3 also preferred egalitarian relationships when they were younger. The partners that they dreamt of united ethics and desire, as it can be seen through words such as “nice” or “treats you well” for the former and “attractive” and “brave” for the latter. Moreover, in this case, unlike M2, no deceit is shown toward these ideas. In fact, this contrasts with the peer pressure (e1) young women in M3 describe:

“*Yes, social pressure in general. When someone started dating guys, you felt pressured not to be the last one. There might have been things that you did not like at all but you did not think about them because what most people thought of that person was more important.*” (P12)

All young women in M3 recognize to have felt social pressure and to have fallen into it. As shared by P12, they acknowledge that they felt pressured to start hooking up with boys, even when they did not really like what was going on, because they were more concerned about fitting in. In addition, through this extract we can recognize another common characteristic of young women in M3, regarding their partner and relationship choices (e2). Likewise, P12 acknowledges to have chosen the partners and relationships which were valued in her social groups and looked right to her friends. In a similar sense P11 explains:

“*You were pressured to hook up with one of those [a popular guy], but really with anyone. It had to be a jerk. He didn’t have to be the coolest. Hooking up with someone of that style was enough.* [My friends] *They never pressured me to hook up with a nice guy*.” (P11)

In this extract P11 explains how you had to choose a partner that was socially valued in order to be socially valued too, and how her friends were the ones pushing her to do it. However, from her words one can see that she resents this behavior, because she states that it never targeted the nice boy. Rather, she says she was pushed to engage in coerced relationships (e3). In Fact, when talking about those, the young women in M3 display negative feelings toward coercive partners and/or coerced relationships. As P11 shares:

“*I did not like the boy, or what was happening, I just hooked up with him to take away that pressure, but I didn’t like it. Those are guys who take advantage of these pressures. They do not care about you. I did not have a good time. I did what I did not because I wanted to, but because I felt I had to. At some point I came to want to do it, but I wanted more to stop being the one that did not hook up, than hooking up itself* [.] *I would say that I did not feel pleasure. Once I hooked up with a guy and, on the next day, I felt so disgusting. And at that moment I was disgusted, that’s why it was the last time. I thought that it changed the way they saw me, I wanted to think that they saw me as being at their level.*” (P11)

As seen in this extract, young women in M3 are very critical about their past coerced relationships and the partners they used to choose. They acknowledge feeling disgust, doing it without really feeling to and giving greater consideration to social status. However, the rejection present in their statements and their critical thinking allows them to recognize that they engaged in fake narratives. Nevertheless, they also explain that they do not do so anymore, because they have broken with the social pressures that pushed them to such behavior. As P12 explains:

“*When I hooked up with a guy I did not like, I told them* [my friends] *it had been cool, and it really hadn’t. Not anymore. I am very honest with myself, if I do not like someone, I am not with him. I do not have to lie; I don’t make things complicated*.” (P12)

In this vein, regarding the presence of fake narratives, young women in M3 do not try to justify themselves or share coerced relationships and partners as exciting anymore, in order to meet social expectations. On the contrary, breaking with the social pressure and acknowledging the lack of pleasure in the relationships they had allows them to understand that these are not what they really want and to stop the circle of lies. P12 admits not feeling the need to lie anymore because she just does not engage with partners she does not like. P11 recognizes not feeling pleasure, rather disgust. This critical reflection allowed her to unmask the truth behind coerced relationships and free herself from the peer pressure that had led her to choose those relationships and partners. Partners who she acknowledges did not care about her but took advantage of the situation.

Taking all the above into account, one clearly sees a common characteristic of M3 young women: *liberating transformation* (e5). Participants in M3 have come to reject past relationships based on pressures and now look for relationships and partners in which there is love, freedom, and attraction. For this reason, participants in M3 state to now prefer *egalitarian relationships and partners* (e6) that unite ethics and desire:

“*My current relationship* [is the best]. *This relationship brings me positive things in all aspects. It adds. it is a different relationship from the rest, it is something I had not felt before by anyone else, it is special. it’s healthy, I have full confidence in my partner, like he has in me. it’s not routineer, but it’s very stable; I laugh a lot, even on bad days. it’s a super nice, stable and real relationship. My most exciting relationship is also this one, there is always novelty, plans for the future, fun, sexual passion. I think they coincide* [the best and the most exciting] *because there is a balance between physical and spiritual attraction. Everything is connected and compensated*.” (p12)

As P12 explains, she now has a relationship that pleases her in every way. It is both her best experience and her most exciting one, because as she explains there is both the physical attraction and the spiritual connection. In a similar way, P11 explains:

“[my best relationship] *is the last one, because it was not with any pressure, because it was what I wanted. It was not with one of those guys who takes advantage of that* [coercion, social pressure]. *You have freed yourself from that* [coercion, social pressure] *and you are well, and you can enjoy. You enjoy really choosing. The most exciting [relationship] is that one too. I was excited because I liked him, and I felt he liked me*.” (p11)

These two last testimonies reflect the transformation that M3 young women have experienced regarding sexual affective relationships, partners, and pleasure. Even if they once fell for them, they now clearly reject past relationships based on pressures and choose egalitarian relationships and partners because they unite ethics and desire. Therefore, even though they initially subjected pleasure to coercion and power, they have seen that this is greater when it is associated with falling in love and freedom. They have taken back their *right to pleasure of falling in love* (e7).

## Discussion

The aim of this research is to shed new light on the influence of the CDD on the pleasure that young women feel or felt in their intimate relationships. The results found in this paper have led to three different models of women (in the sense of the Weberian ideal types) regarding the effects of the CDD on their partner and relationship choice and its relation to the pleasure they feel.

M1, as teenagers, rejected the pressure to engage in casual sex with those portrayed by the CDD as the “cool boys” and sought for egalitarian relationships and partners in which they united both ethics and desire. Some studies point out that frequent communication with parents regarding sexual issues significantly reduces falling into peer pressure (van de Bongardt et al., 2014). As well social interactions have proven to be a key element in rejecting the pressures of the CDD (Puigvert Mallart et al., 2019). However, further research is needed in order to provide more knowledge about the factors that allow girls and women to reject the CDD and freely choose egalitarian relationships.

Currently, M1 participants still link pleasure to their romantic relationships. They describe their partners as being supportive and good, but also attractive and passionate. They feel that their relationships are what they have always wanted, with sexual desire being an important part of them. Therefore, pleasure relies in the relationships they have built and the connection they feel. These results are consistent with other studies reporting greater sexual satisfaction in romantic relationships (Armstrong et al., 2012; Barnett and Melugin, 2016).

Moreover, the narratives of participants in M1 challenge the idea of the downside of long-term relationships having worse outcomes for women than hooking up (Armstrong et al., 2010), as all participants in romantic relationships (love-based) reported high satisfaction with their relationships. Indeed, these findings support the idea that the positive or negative outcomes of a relationship are not in its duration, but in the partner of choice. These findings are more aligned with those of Carlson and Soller (2019), which found sexual empowerment and sexual well-being in egalitarian relationships, led by increased communication within the couple. The narratives of participants in M1 also contribute to the empowerment of all those girls and women who freely decide to never engage with men portrayed as more attractive through the CDD because of being violent. In line with previous research by Puigvert Mallart et al. (2019), the self-interrogation that this girls undergo about who they want to be and with whom they choose to have sexual relationships allows them to break the association between violence and attraction, and to associate this later feeling to partners that respect them and feel too passionate about them. Furthermore, they prove that one does not need to suffer the negative consequences of coerced relationships to reject them.

Conversely to participants in M1, M2 participants link pleasure to elements of the CDD. In their narratives, directly or indirectly, they describe peer pressure to hook up with the bad boys. M2 participants’ attitudes toward casual relationships are consistent with Suleiman and Deardorff (2015), which found that the majority of participants in their study mentioned their choice of relationship being influenced by their peers. In addition, young women in M2 indicate having engaged in such relationships for status matters. They felt that conveying which was expected from them would make them more socially valued by their peers. This behavior can be explained when girls and/or young women fall victims of the “upward mobility mirage” (Oliver, 2010–2012), which results from the CDD present in interactions with peers. It is also consistent with other studies (Pedersen et al., 2017), in which participants, through their narratives, share that having wild experiences to recount to friends is important to them. In addition, the fact that all young women in M2 recall engaging in sexual–affective relationships that they did not enjoy or choosing partners who they did not like is also in accordance with studies (Montes et al., 2016, 2017) showing that students experiencing peer pressure to engage in casual relationships seem more susceptible to adverse outcomes related to hooking up.

Nevertheless, young women in M2 also had good memories about these relationships, even if vaguer. These results would be consistent with studies reporting that pleasure in casual relationships often lies beyond the sexual encounter itself (Farvid and Braun, 2017; Pedersen et al., 2017). Rather, these studies show that pleasure in these encounters relies on elements as the “hunt,” status, or sharing the story with friends; all these elements being consistent with the CDD. For instance, participants in Pedersen et al. (2017) recounted their “wild experiences” as being fun, but provided ambivalent reports of these encounters. Thus, by including in the analysis the existence of the CDD, the present study provides new insights on the fact that girls and women under the pressures of the CDD engage in relationships that are not pleasurable *per se*. This is in line with studies from the field of socioneuroscience which explain how the CDD can shape the neural networks of some women and lead them to feel attraction toward these men and relationships, even though they can be aware at the same time that they do not feel pleasure in them. These finding can also contribute to explain why some women feel regret (Campbell, 2008) or disgust (Al-Shawaf et al., 2018; Kennair et al., 2018) after hooking up, by evidencing the contradictions between what they think of these relationships because of the CDD and what they actually experience in them. Moreover, the current study challenges those studies that explain these feelings of regret as a consequence of social double-standards that punish women, and not men, for being in casual relationships (Armstrong et al., 2010; Snapp et al., 2015; Rodrigue and Fernet, 2016; Farvid and Braun, 2017; Uecker and Martinez, 2017). In this vein, it provides evidence on how their choices are a consequence of the CDD, which in turn drives them to have behaviors that they do not fully consciously decide and thus, they later regret.

Furthermore, evidence in the current study supports that hook-ups do not inherently lead to negative outcomes. Rather it points out that they have negative effects on women when they are the consequence of the CDD, which is consistent with previous investigations (Valls et al., 2008; Puigvert et al., 2019).

In addition, the present study also provides evidence about how engaging in coerced relationships can have long-term effects in later relationships and partner choice. Participants in this study classified as M2 currently report to prefer egalitarian relationships, while they still think that hook-ups were more exciting. This drives them to a double standards cul-de-sac: while egalitarian relationships are now seen as more convenient, hook-ups with the “bad boys” still are more exciting (Gómez, 2015). This apparent contradiction could be explained at the light of results found by Racionero-Plaza et al. (2018), which show that violent sexual–affective relationships could include feelings of attraction and desire. According to the authors, this behavior would be influenced and triggered by the consequence of storing in their memory coerced situations as desirable, because of the CDD, and would, in turn, set a frame of reference infused of coercive elements for future relationships (Racionero-Plaza et al., 2018). Findings from the field of socioneuroscience also support this idea and provide an explanation of why it happens: in the same schemata and as a consequence of the CDD, there are stored memories of aggression in an intimate relationship (what happened) and attraction toward the aggressor.

Therefore, the current study points out how the CDD might be responsible for leading adolescents to engage in pleasureless and coerced relationships, where pleasure lays outside the relationship itself. Moreover, the results presented in this paper also challenge the idea that bad hook-ups do not have negative long-term effects on women’s relationships (Armstrong et al., 2010; Farvid and Braun, 2017). As well, it offers an explanation to the mixed-results found in the association between well-being and hooking-up (Vrangalova, 2015).

Finally, M3 participants explain how they fell into the pressures of the CDD and engaged in relationships with the bad boys for external reasons to the relationships, just as M2 participants (Oliver, 2010–2012; Gómez, 2015). However, conversely to those in M2, M3 participants fully acknowledge the lack of pleasure in those relationships, as well as the fact that they used to share them as exciting to meet social expectations. The capacity of these young women to break with the pressures of the CDD and transform their relationships is consistent with the analysis of Puigvert Mallart et al. (2019), who explain that critical awareness about the influence of CDD is key to dissociate attraction from violent partners and relationships. Indeed, all young women in M3 who undergo the process of rejecting the CDD regain control of their choices and preferences and transform the type of relationships they prefer.

In addition, these findings also contribute to explain the fact that women in egalitarian relationships report pleasure to be greater in such relationships, just as participants in M1. However, participants in M3 also contribute to challenge the pressures that women in M1 suffer when they are told that they prefer their egalitarian relationships because they do not know other relationships than those. Indeed, women in M3 provide first-hand narratives that unveil the lack of pleasure in coerced relationships and recognize how sexual pleasure can only be found with an egalitarian partner that does not treat you with contempt. Indeed, their narratives shed new light on aspects such as why the pleasure relies in external factors of the relationship [i.e., the hunt, status, feeling wild, etc. (Farvid and Braun, 2017; Pedersen et al., 2017)] and provide an explanation to why adolescents and young adults allege to engage in such relationships for the sex, while they also report the sex to be less physically pleasurable (Farvid and Braun, 2017). They also provide an alternative answer to why women can easily share the details about the lack of pleasure in coerced relationships but not the positive ones: challenging Farvid (2014) hypothesis, this would not be because they lack to resources to narrate such pleasures, but simply because these experiences were never truly pleasurable.

In addition, M3 participants support that egalitarian romantic relationships can be sexually empowering and satisfactory (Carlson and Soller, 2019). According to these participants, their egalitarian relationships are of higher quality (Paik, 2010) and more pleasurable, not only regarding orgasm (Armstrong et al., 2012), but because they unite in the same person passion and love (Gómez, 2015). These findings bring forward the idea that pleasure is closely associated with falling in love and challenge once again the idea of love being a damaging force (Lelaurain et al., 2018).

### Limitations

The current research aimed at exploring pleasure taking into account the presence of the CDD. Our findings show how this new approach provides new answers for questions and behaviors that remained unexplained. However, because of its exploratory nature, the current study does not provide an in-depth explanation of the elements underlying the association between pleasure and the CDD, for which further research is needed.

Another limitation of the study is that participants shared their own narratives. Therefore, it must be taken into account that some questions were partially answered or that some tried to respond in line with what they consider to be more socially valued. This limitation was addressed by asking the same questions from different angles, which allowed us to detect inconsistencies and ask for clarifications. Nevertheless, the possibility of answers not being fully representative of their experiences cannot be rejected.

### Further Research

The present study has unveiled new elements that should be considered in future researches. One of these elements of study refers to peer pressure under the CDD, in order to gain new insights on which factors put girls and women at risk of falling into its pressures and which factors can be considered protective. In this line, young women in M1 can provide new valuable knowledge.

Another element worth considering is the role of fake narratives in associating pleasure to coerced relationships. Providing new evidence of why girls and women engage in these practices, as well as to contrast what they experience with what they share would allow to provide further evidence supporting the lack of pleasure in coerced relationships. Young women in M2 and M3 could provide first-hand knowledge of this process. Moreover, the participation of young women in M2 in such a study under the frame of communicative methodology would provide them with scientific knowledge that could allow them to challenge their perceptions and engagement in coercive relationships.

Finally, further focus on egalitarian relationships will provide new evidence of which elements make these relationships pleasurable, contributing to a body of knowledge in which romantic relationships challenge the dichotomy between exciting relationships and convenient ones by uniting in the same person passion and love.

## Conclusion

The current research is one of the many studies now contributing to further increase the social impact of psychology by providing new evidence regarding pleasure in sexual–affective relationships. Accounting for the presence of a CDD, this research has shown how peer pressure can lead to coerced relationships which completely lack pleasure but are perceived as pleasurable because of elements that lie outside of them and that are socially constructed. Unveiling this fact has allowed us to see how those young women who reject the pressures of the CDD (M1) choose egalitarian relationships in which there is love and excitement. Pleasure is an important part of the relationship they have built; they have not given up the pleasure of falling in love.

On the contrary, those young women who fall into the pressures of the coercive discourse (M2) report ambivalent feelings about the relationships in which they used to engage: they signal that they started because they felt they had to and they describe experiencing negative situations; however, they still describe them as exciting because pleasure is subject to the elements of the CDD. Far from being isolated experiences with no further effects, this study shows how such behavior steals from women their right to the pleasure of falling in love, as it leads them to assume that egalitarian relationships even if convenient are boring, and that excitement lies elsewhere.

Nevertheless, our findings also suggest that the negative effect of falling into the pressures of the CDD can be overcome. This transformation lies in the rejection of coerced relationships by recognizing the lack of pleasure in them, the lack of truth in what was shared by friends (fake narratives) and the lack of freedom in those choices. In this vein, young women in M3 show how the right to the pleasure of falling in love can be taken back, since this transformation not only allows to unveil the negative truth behind coerced relationships, but also reveals how romantic relationships (long or short) are intrinsically free and satisfactory. As a M3, P11, stated:

“*Those who still look for hook-ups, want the next one to be even worse; it’s always like this.*

Those who want to settle in, look for nice guys who they think will accept them.

*Those who transform themselves, look for the prince*.”

As women, we thus vindicate our right to the pleasure of falling in love.

## Data Availability Statement

The datasets generated for this study are available on request to the corresponding author.

## Ethics Statement

The studies involving human participants were reviewed and approved by the Ethics Board of the Community of Researchers on Excellence for All (CREA). The patients/participants provided their written informed consent to participate in this study. Written informed consent was obtained from the individual(s) for the publication of any potentially identifiable images or data included in this article.

## Author Contributions

LP was the IP of the research line from which this article derives. ET-G was responsible for the article’s design and writing. AK and ET-G participated in some of the fieldwork. EA collaborated in the current line of research with LP and in the design of the article with ET-G. LP and ET-G participated in the analysis. AK and ET-G co-authored a communication on this topic at an international feminist conference.

## Conflict of Interest

The authors declare that the research was conducted in the absence of any commercial or financial relationships that could be construed as a potential conflict of interest.
